# Why and how to spare the hippocampus during brain radiotherapy: the developing role of hippocampal avoidance in cranial radiotherapy

**DOI:** 10.1186/1748-717X-9-139

**Published:** 2014-06-16

**Authors:** Tomas Kazda, Radim Jancalek, Petr Pospisil, Ondrej Sevela, Tomas Prochazka, Miroslav Vrzal, Petr Burkon, Marek Slavik, Ludmila Hynkova, Pavel Slampa, Nadia N Laack

**Affiliations:** 1 Department of Radiation Oncology, Faculty of Medicine, Masaryk University and Masaryk Memorial Cancer Institute, Zluty kopec 7, 656 53 Brno, Czech Republic; 2 International Clinical Research Center, St. Anne’s University Hospital Brno, Pekarska 53, 656 91 Brno, Czech Republic; 3 Department of Neurosurgery, Faculty of Medicine, Masaryk University and St. Anne’s University Hospital Brno, Pekarska53, 656 91 Brno, Czech Republic; 4 Department of Medical Physics, Masaryk Memorial Cancer Institute, Zluty kopec 7, 656 53 Brno, Czech Republic; 5 Department of Radiation Oncology, Mayo Clinic, 200 First Street SW, Rochester, MN 55905, USA

**Keywords:** Hippocampus, Hippocampal sparing, Hippocampal avoiding radiotherapy, Brain radiotherapy, Feasibility study, Planning study

## Abstract

The goal of this review is to summarize the rationale for and feasibility of hippocampal sparing techniques during brain irradiation. Radiotherapy is the most effective non-surgical treatment of brain tumors and with the improvement in overall survival for these patients over the last few decades, there is an effort to minimize potential adverse effects leading to possible worsening in quality of life, especially worsening of neurocognitive function. The hippocampus and associated limbic system have long been known to be important in memory formation and pre-clinical models show loss of hippocampal stem cells with radiation as well as changes in architecture and function of mature neurons. Cognitive outcomes in clinical studies are beginning to provide evidence of cognitive effects associated with hippocampal dose and the cognitive benefits of hippocampal sparing. Numerous feasibility planning studies support the feasibility of using modern radiotherapy systems for hippocampal sparing during brain irradiation. Although results of the ongoing phase II and phase III studies are needed to confirm the benefit of hippocampal sparing brain radiotherapy on neurocognitive function, it is now technically and dosimetrically feasible to create hippocampal sparing treatment plans with appropriate irradiation of target volumes. The purpose of this review is to provide a brief overview of studies that provide a rationale for hippocampal avoidance and provide summary of published feasibility studies in order to help clinicians prepare for clinical usage of these complex and challenging techniques.

## Background

Both primary and secondary brain tumors (BT) represent a significant public health problem. An increasing incidence in primary brain tumors (PBT) as well as brain metastasis (BM) has been documented over recent years. In 2014, more than 24,000 new PBT are estimated to be diagnosed in the United States [1]. Moreover, about 1,4 million new solid tumor cases of all histological origin are diagnosed each year in the United States and approximately 30% of them develop BM [1]. Therefore, management of BT is an increasingly important component of cancer therapy [2].

Radiotherapy is an important modality in the treatment of BT. Radiotherapy remains the standard treatment for vast majority of high-grade or malignant brain tumors and plays an integral role in treatment of many low-grade and benign primary brain tumors. However, concerns regarding neurocognitive toxicity after radiotherapy in patients with benign or low-grade tumors make the timing of treatment controversial [3].

Historically, radiotherapy was also a mainstay of treatment for BM. With improved survival and increased awareness of the cognitive effects of WBRT, the role of WBRT in BM has come under question [4]. Because of these concerns there has been a trend towards increased reliance on focal treatments such surgery and stereotactic radiosurgery (SRS) [5]. However, achieving whole brain control is associated with improved survival and preserved neurocognitive domains with exception of memory function, especially recall and delayed recall [6]. Thus, understanding the risk of brain tumor recurrence at distant sites of brain is important in counseling patients regarding the risks and benefits of WBRT. Patients with single BM and no extracranial metastases are at low risk for in-brain recurrence and omitting early WBRT because of the risk of intermediate and late adverse effects (AE) can be safely done as long as the patient commits to regular imaging [7]. Conversely, patients with progressive systemic disease are at a higher risk for distant brain failure and likely benefit from the addition of WBRT despite possible late complications [8].

For most malignant adult PBT and BM, radiotherapy prolongs survival but is rarely curative. Thus, emphasis on minimizing the AE of treatment is becoming one of the most important factors in the treatment. Recently, more attention has been paid to symptom related outcomes of care, especially to neurocognitive function (NCF) and quality of life (QoL) [9-11]. With improvements in radiotherapy systems technology, it is now possible to modify treatment plans to selectively spare structures that may contribute to decreased QoL and NCF. In order to achieve this aim, it is important to determine appropriate end-points primarily in relation to the ongoing randomized clinical trials as resources for future treatment guidelines [12].

Decline in NCF as an iatrogenic side effect of brain irradiation is well-known [13]. The mechanism of radiation injury is complex and multi-factorial. In the past, cognitive decline after radiotherapy was believed to be a late effect of treatment mediated through microvascular changes and neuroglial loss. However, there is increasing evidence for acute and subacute cognitive changes after radiotherapy that appear to be mediated through the neurogenic zones including the hippocampus. Preclinical evidence supports the concept of hippocampal radiation injury as a mediator of subsequent AE, most notably in memory-related domains of neurocognition [14]. Retrospective clinical reports as well as early results of prospective trials support the role of hippocampus in early changes in cognitive function after radiotherapy [11]. The purpose of this review is to provide a brief overview of studies that provide a rationale for hippocampal avoidance (HA) and provide summary of published feasibility studies in order to help clinicians prepare for clinical usage of these complex and challenging techniques.

### Hippocampus and radiation injury

The hippocampus is a paired brain structure, located in the ventromedial part of the temporal lobes, laying lateral to the temporal horn of the lateral ventricle. The hippocampus is composed of the dentate gyrus and the cornu ammonis regions and belongs to the limbic system. Its main role in brain function is cooperation in learning, consolidation and retrieval of information and it is also essential for formation of new memories [15]. Bilateral and unilateral radiation injury of the hippocampus is known to alter learning and memory formation [16]. Complete pathophysiologic explanation of all these processes is still lacking; nevertheless, the role of neurogenesis seems to be one of the most compelling [17].

Mitotically active neural stem cells (NSCs) are located in different parts of brain, namely in the subependymal zone and in the subgranular zone of the dentate gyrus, wherefrom they migrate into the granular cell layer of hippocampus [18]. The hippocampal subgranular zone is a critical neurologic center for learning and memory [19]. NSCs have typical features of the stem cells. They are capable of both self-renewal and generating new differentiated cells [20]. Neurogenesis is a complicated process with integration of many regulatory cells as astrocytes or endothelial cells with coordinate evolution of neural precursor cells together with each other in a specific neurogenic microenvironment called “niche” [21,22].

Multiple preclinical studies support the hypothesis of hippocampus-mediated cognitive dysfunction [23-30]. In vivo animal studies demonstrate sensitivity of these NSCs to ionizing radiation. Apoptosis of NSCs after ionizing radiation was first described in the subependymal zone in the young adult rat. After single x-ray doses of 5 or 30 Gy, apoptosis peaked 6 hours after irradiation [23]. Several years later, postradiation apoptosis was observed also in the rats’ dentate gyrus after exposure to single 10 Gy dose [24]. Decline in neurogenesis was associated with cognitive impairment in rodent models for both single and fractionated brain irradiation [14,25]. Mizumatsu et al. irradiated the whole brain of experimental mice with various single doses and used immunohistochemical staining methods for detection of apoptosis as well as numbers of proliferating cells and immature neurons in the subgranular zone of the hippocampal dentate gyrus. Dose-dependent apoptosis was observed and peaked 12 hours after irradiation followed by subsequent reduction in amount of proliferating cells in subgranular zone [27]. Changes in neurogenesis were associated with an inflammatory response as validated by detection of activated microglia cells [29]. Moreover, administration of anti-inflammatory agents such as ramipril and indomethacin can mitigate radiation-induced cognitive impairment in rodents suggesting the inflammatory response is important in mediating the effects of radiotherapy [30].

However, these and other mechanisms of radiation effects on neurogenesis do not completely describe the radiobiology of the hippocampus [31]. More recent in vitro and in vivo research reveals other important radiation induced hippocampal changes which may also influence cognition [32]. Investigators at University of California at Irvine, CA, USA have optimized a SYBR green based assay to study the effects of low dose RT before changes are visible radiographically. Even a dose of 2 Gy delivered to human NSCs leads to decreased numbers of cells undergoing neuronal differentiation after irradiation [33]. Additional work from the same group suggests the mechanism of radiation-induced inhibition of neurogenesis may be mediated through oxidative stress [34].

Although significant pre-clinical data supports a decrease in NSC number and function after radiotherapy, changes in neuronal architecture, as recently described by Parihar and Limoli in measurements of micromorphometric parameters in mice following cranial irradiation by 1 and 10 Gy, may also be important in mediating the effects of radiotherapy [35]. Dose-dependent reduction of dendritic branching, length and area were described as well as the reduction of immature filopodia as compared with mature spine morphology of dendritic segments. These postradiation changes correlated with alterations in synaptic protein production that were noted up to 1 month after brain irradiation [35]. These types of changes are likely to be equally important as disruption of neurogenesis in eliciting cognitive decline after radiotherapy.

Recent studies provide dose–response data and estimation of clonogenic survival fraction of human NSCs after brain irradiation. This data is important from a radiation oncology point of view primarily for determination of pertinent treatment planning recommended dose constraints.

In QUANTEC analysis, the normal brain α/β value has been established to be 2.9 [36]. For the hippocampal region, most authors use the α/β ratio in range from 2 to 3 [37]. However, other authors work with the α/β value for NSCs compartments equal to 10 [38,39] using a general value established for stem cells [40]. Some authors use α/β ratio 10 for the true hippocampus and an α/β value 2 for the whole hippocampus planning-at-risk volume illustrating the lack of consensus regarding the optimal model of radiation sensitivity. However, preclinical evidence suggests doses as low as 2 Gy to result in apoptosis of neurogenic stem cells supporting a no-shoulder dose-response [29,33]. Several other studies have measured altered survival and proliferation using a range of metabolic and SYBR green based assays. These studies revealed that doses of as low as 2 Gy reduced survival by over 50% [33]. Thus, accumulating preclinical data indicate that neurocognitive dysfunction manifests at much lower doses (<10 Gy) than previously expected [34].

### Clinical evidence for hippocampal sparing

In addition to preclinical evidence, retrospective clinical reports also suggest the hippocampal region may play a role in NCF decline after radiotherapy. Children with brain tumors treated on prospective clinical trials that included planned neurocognitive assessments were evaluated with neurocognitive studies up to 5 years after radiotherapy. Mean doses of 45 Gy or higher to the left temporal lobes were associated with significant declines in longitudinal IQ [41,42]. The relationship between hippocampal dose level and the risk of subsequent NCF impairment was described in the group of patients with adult low-grade gliomas; NCF was assessed at the baseline and at 18 months follow-up for conventionally treated patients. Biologically equivalent dose greater than 7.3 Gy (equivalent dose in 2-Gy fractions) applied to 40% of hippocampal volume was associated with long-term NCF impairment, especially in list-learning delayed recall [43]. Recently, results of the first prospective phase II study (RTOG 0933) of HA in BM patients suggest a reduction in risk of cognitive dysfunction with HA [11]. Primary cognitive outcome was delayed recall at 4 months as measured by the Hopkins Verbal Learning Test for patients with WBRT comparing with those with HA WBRT. Results were compared to historical control group. Only 7% of patients experienced decline in memory compared to 30% of patients in the historical cohort (p = 0.0003). QoL was evaluated as well and was preserved up to 6 months follow-up. Based on these results, RTOG is planning a phase III randomized trial of prophylactic cranial irradiation with or without hippocampal sparing for small cell lung cancer patients (RTOG 1317).

### Hippocampus-sparing: feasibility studies

**Figure 1:**
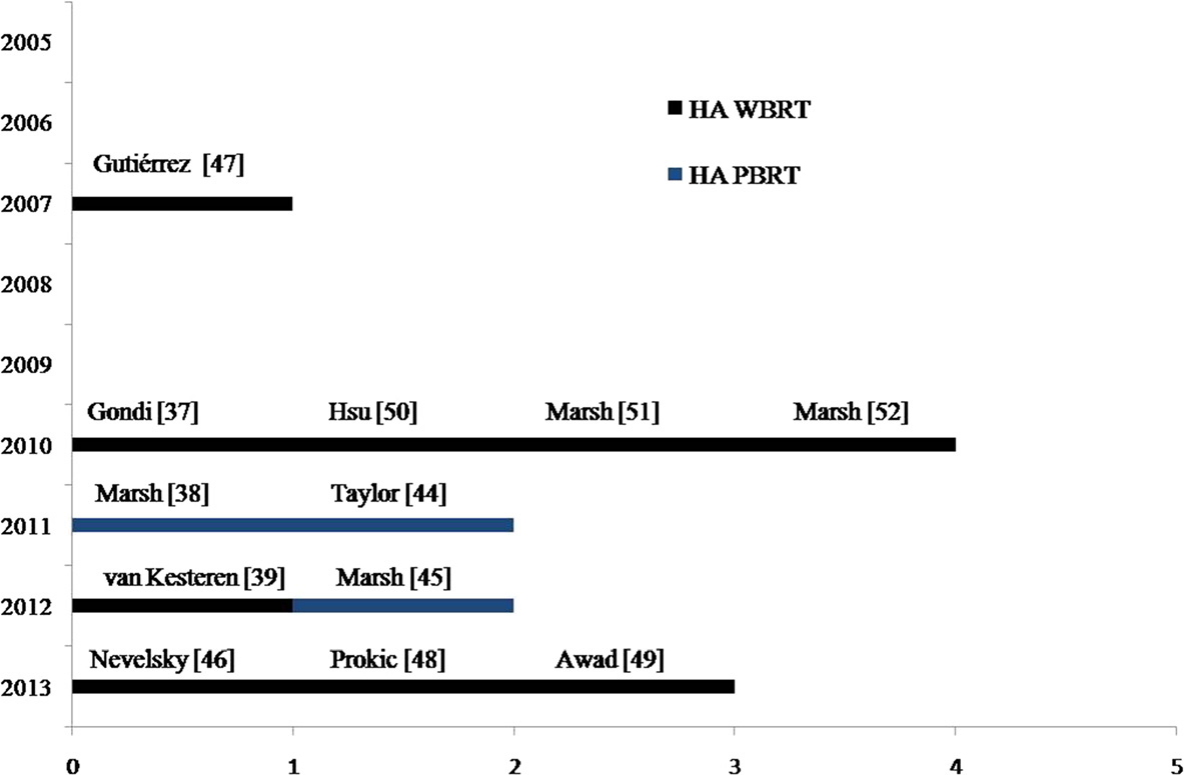
**Overview of recently published radiotherapy planning studies dealing with hippocampal avoidance. **Only studies with more than 9 patients included [44].

Although evidence is mounting in regards to the importance of the hippocampus in mediating cognitive changes after radiotherapy, it has only been with recent technologic advances that the feasibility of a meaningful reduction in hippocampal dose while maintaining acceptable tumor control probability has been established. Below we review the feasibility studies evaluating HA for PBT and BM (Figure 1).

### Primary brain tumors: HA feasibility studies

Complex intensity modulated radiotherapy (IMRT) plans were developed to evaluate the feasibility of sparing contralateral and bilateral hippocampi in glioma patients [38]. In “sparing” plans for the hemispheric HGG cases, it was possible to reduce the mean physical dose to the contralateral hippocampus planning-at-risk volume by 56.8% compared to standard treatment plan prepared without prospective sparing of the hippocampus (15.8 Gy vs. 36.6 Gy). In addition, more central location of PBT enables sparing of both hippocampi as documented by mean physical dose reduction by more than a third (16.8 Gy in sparing vs. 25.6 Gy in standard plan) [38]. Based on previous preclinical data, even low doses can result in NSCs apoptosis, but assuming that hippocampus is more parallel than serial organ, a reduction of median hippocampal dose may reduce NCF impairment even if no unequivocal cut-off dose threshold is known. Unfortunately, reported dose reduction is related to hippocampal planning-at-risk volumes, which were created by 3 mm expansion of hippocampi, so it is not possible to compare these results with other published studies.

PBT are frequent diagnosis in children in whom minimization of subsequent late AE is even more important. In a evaluating HA in pediatric gliomas, the NSCs compartments, the limbic circuit and the whole hippocampus were recognized as organ at risk (OARs) and included in experimental treatment plans [45]. For each RT plan the biological equivalent doses were calculated providing more radiobiologically exact comparison of doses in OARs. In all cases (10 different PBT), experimental plans significantly reduce both mean physical dose (by 56.0%) and mean biological equivalent doses (by 52.1%) delivered to the study OARs in comparison to plans without any effort to spare these structures. As might be expected, greatest hippocampal sparing was seen in hemispheric gliomas whereas worse results were observed for diffuse tumors where whole ventricular RT was indicated.

### Brain metastases: HA feasibility studies

Many planning studies have shown the dosimetric feasibility of HA WBRT using different radiotherapy systems as linear accelerator (LINAC) based IMRT, helical tomotherapy or volumetric modulated arc therapy (VMAT). In addition, HA brain irradiation is also possible using Elekta IMRT step and shoot systems [46].

The pioneer study was performed in 2007 by Gutiérrez et al., who tested feasibility of HA WBRT with simultaneous integrated boost (SIB) to BM in experimental radiotherapy plans using helical tomotherapy [47]. Regardless a different setting of treatment plans (pitch and field width), they described no significant difference in hippocampal doses. Authors concluded that it is possible to create combined plans with homogeneous whole brain dose distribution equivalent to conventional WBRT, while conformal HA and radiosurgically equivalent dose boosting to individual metastases.

Mean dose guidelines for HA WBRT were first published by Gondi [37]. HA plans were compared with standard WBRT ones where a homogenous dose 30 Gy was applied to the whole brain including hippocampus. For HA plans, the median hippocampal dose was achieved 5.5 Gy (D_max_ 12.8 Gy) and 7.8 Gy (D_max_ 15.3 Gy) for helical tomotherapy and LINAC based RT, respectively. These dose reductions have been considered a reference for other subsequent planning studies.

Because of higher availability of stereotactic systems, recent trends are to combine WBRT with stereotactic boosting to BM and thus improve local control. Also in this sequential concept, it is possible to spare hippocampus in both parts of treatment: HA WBRT and subsequent HA SRS boost. Moreover, using IMRT, it is possible to integrate boosting into the first WBRT part in concept of SIB. Comparing this approach with classical sequential concept (WBRT + stereotactic radiotherapy), the SIB is more effective in lowering doses to the hippocampus for patients with up to 8 metastases [48].

Although multiple techniques allow HA WBRT, treatment time can vary significantly depending on the technique. Using VMAT for HA WBRT with SIB for melanoma brain metastases, the average beam-on time was achieved 3.6 min while abide the RTOG 0933 feasibility DV constraints [49]. Arc based delivery times are generally faster than conventional IMRT. VMAT was 3.5 times faster than classical IMRT techniques as discussed in other one planning study focused on HA WBRT published last year [46,50].

Similarly as for BM treatment, HA WBRT technique can be used for prophylactic cranial irradiation (PCI) where are NCF preserving approaches even more justified. Comparisons of limbic sparing experimental plans were conducted in 11 patients indicated for WBRT and for PCI. Similar reduction of hippocampal biological equivalent doses was achieved for both of these clinical situations [51,52]. These results are not surprising considering the fact, that PCI differ from WBRT only in terms of fractionation and that the standard radiotherapy technique is similar.

Comparison of treatment planning results with other studies is summarized in Table 1.

**Table 1:** Hippocampal dose-volume constraints and achieved doses in representative HA WBRT planning studies

**Author, year**	**Clinical situation**	**RT system**	**No.**	**Fractionation**	**Hippocampal constraints**		**Hippocampal doses**			**α/β**	
					**D**_**max**_		**D**_**max**_	**D**_**mean**_	**D**_**median**_		
Gutierrez, 2007 [47]	WBRT	HT	10	15 × 2.15 Gy	6 Gy		-	5.86 Gy_2_	5.34 Gy_2_	2	
Gondi, 2010 [37]	WBRT	HT	5	10 × 3.0 Gy	6 Gy	3 Gy ≤ 20%	12.8 Gy	-	5.5 Gy	2	
		LINAC			11 Gy	9 Gy ≤ 40%	15.3 Gy		7.8 Gy	2	
Hsu, 2010 [50]	WBRT + SIB	LINAC	10	15 × 2.15 Gy (SIB á 4.2 Gy)	-	D_mean_ < 6 Gy_2_	-	5.23 Gy_2_	-	2	
Marsh, 2010 [51]	PCI	HT	11	15 × 2.0 Gy	15 Gy		-	12.5 Gy	-	-	
	WBRT		11	14 × 2.5 Gy	15 Gy			14.3 Gy			
Marsh, 2010 [52]	PCI	HT	10	15 × 2.0 Gy			-	11.5 Gy	-	-	
	WBRT		10	14 × 2.5 Gy	-	-	-	11.8 Gy	-	-	
Van Kesteren, 2012 [39]	WBRT	LINAC 3D-CRT	10	12 × 2.5 Gy			13.5 Gy	6Gy	-	10	
Nevelsky, 2013 [46]	WBRT	LINAC IMRT	10	10 × 3.0 Gy	16 Gy	D_100%_ < 9 Gy	14.35 Gy	-	-	-	
Awad, 2013 [49]	WBRT + SIB	VMAT RA	30	5-15fx	-		32.2 Gy	20.4 Gy	21.9 Gy	-	
Prokic, 2013 [48]	WBRT + SIB	VMAT RA	10	12 × 2.5 Gy BM 12 × 4.25	-		12.33 Gy (D_2%_)	7.55 Gy	7.15 Gy	H 2	BM 10
	WBRT + FSRT	VMAT RA	10	12 × 2.5 Gy + FSRT 2 × 9 Gy	-		15.82 Gy (D_2%_)	9.8 Gy	9.34 Gy	H 2	BM 10

3D-CRT: three dimensional conformal radiotherapy; RA: Rapid Arc; HT: helical tomotherapy; FSRT: fractionated stereotactic radiotherapy; D_max_: maximal dose; D_mean_: mean dose; D_100%_: dose in 100% of volume; D_2%_: dose in 2% of volume; D_median_: median dose; H: hippocampus.

### How to spare hippocampus

Because of hippocampal anatomic shape and central brain location, it can be a challenge to create appropriate HA treatment plan for irradiation of both PBT and BM. Nevertheless, modern IMRT techniques such as helical tomotherapy or VMAT are able to achieve HA with acceptable target volume coverage and dose homogeneity. Although a variety of treatment techniques are available for HA WBRT, the ability to achieve OAR dose goals varies by technique. In general, helical tomotherapy offered significantly better HA compared to LINAC based IMRT in terms of the mean normalized tissue dose, as well as the median and maximal hippocampal dose. However, despite different technical capabilities of mentioned radiotherapy systems, it can be concluded, that using either helical tomotherapy or LINAC based IMRT is sufficient for sparing not only traditional OARs but also the hippocampus [37]. An effort to minimize hippocampal dose must not lead to irradiation of other brain OARs. This is important especially for gliomas treated by overall higher doses compared with treatment of BM.

In addition, dose constraints are expected to be different for HA WBRT and HA PBRT. For BM, it is possible to sufficiently spare both hippocampi, and dose-volume constraints used in representative HA WBRT planning studies were summarized in Table 1. On the other hand, for HA PBRT, especially in the treatment of hemispheric gliomas, ipsilateral hippocampus is often included in target volumes (and considering much larger doses compared with WBRT). Thus, it is not possible to achieve appropriate dose reduction for ipsilateral hippocampus and only contralateral hippocampus could be considered as OAR. As an example, for HGG the following constraints criteria have been proposed: 0% of the hippocampal volume cannot receive more than 8 Gy in the first phase of treatment (up to 46 Gy) and no more than 4 Gy in the final phase (next 14 Gy to the target volume) [38].

Contouring of target volumes is, as a potential source for systematic error, one of the most important parts of whole radiotherapy planning process. Structure contouring for radiotherapy purpose is sometimes slightly different process comparing with the other medical discipline. Exact volumetric assessing of the whole hippocampus is important especially in basic neurological research [53] as well as in research dealing with diseases connected to hippocampal impairment [54]. On the other hand, only some HA radiotherapy feasibility planning studies defined in detail the process of contouring, almost exclusively with reference to the Radiation Therapy Oncology Group on-line contouring atlas [55]. Authors of this atlas do not contour the entire hippocampus, but are focusing mostly on the subgranular zone as a place of NSCs occurrence. This approach is suggested as a standard for HA WBRT. On the other hand, for HA PBRT, where only the contralateral hippocampus is often spared, it is possible to contour the whole hippocampus irrespective of its NSCs rich part according to a radiation oncologist’s guide to contouring the hippocampus proposed by Chera et al. [56]. Moreover, considering an attempt to spare NCF during brain irradiation, some studies defined OARs even more comprehensively including the whole limbic circuit (whole hippocampus; the rest of limbic circuit comprising the amygdalar complex, the fornix, the cingulum, the cingulated gyrus, and the mammillary bodies) [38,51].

**Figure 2:**
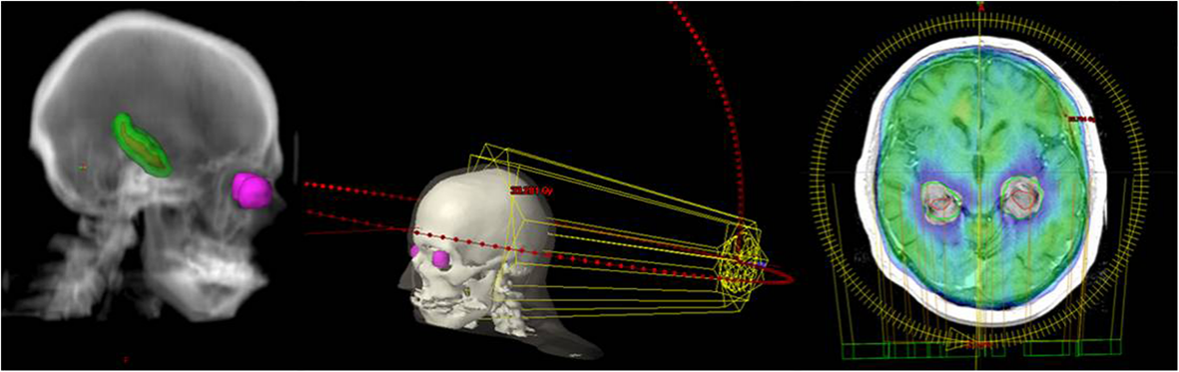
Examples of non-coplanar Arc treatment plan with hippocampal sparing and homogenous dose coverage in the rest of the brain.

Physician’s requirements expressed in terms of dose-volume constraints are best achievable using inverse planning, which enables to set different priority points to different OARs and target volumes and thus it is possible to find compromise in dose coverage for all important treatment structures. As an example, in Table 1 were described parameters for both helical tomotherapy and LINAC based IMRT as presented in a seminal planning study [37]. These set of dose-volume constraints have been used in RTOG 0933 study, a first HA WBRT clinical trial which results were mentioned above [11]. Although methodology of the study enables differences in radiotherapy systems for preparation of a particular treatment plan, plans have to meet the required dosimetric constraints prior to approval for clinical use. Table 2 presents acceptable and unacceptable variations from “per protocol planning” for including particular IMRT treatment plan into RTOG 0933 trial. In our HA planning study, we compare different Arc radiotherapy techniques in order to achieve mentioned constraints. In the setting of non-coplanar beams arrangement, we have observed even higher hippocampal preservation compared to classical coplanar irradiation (not published data) (Figure 2).

**Table 2:** **Acceptable and unacceptable variations from per protocol IMRT treatment planning according to RTOG 0933 trial**[11]

**Treatment component**	**Parameter**	**Per protocol**	**Variation acceptable**	**Unacceptable deviation**
MRI/CT Fusion and Contouring	MRI-CT fusion	No corrections to MRI/CT fusion requested	No corrections to MRI/CT fusion requested	Corrections to MRI/CT fusion requested
	Hippocampal Contouring	≤ 2 mm deviation using the Hausdorff distance*	> 2 and ≤ 7 mm deviation using the Hausdorff distance*	> 7 mm using the Hausdorff distance*
HA WBRT IMRT Planning	PTV	D_2%_ ≤ 37.5 Gy	D_2%_ > 37.5 Gy ≤ 40 Gy	V_30_ < 90%
		D_98%_ ≥ 25 Gy	D_98%_ < 25 Gy	D_2%_ > 40 Gy
	Hippocampus	D_100%_ ≤ 9 Gy	D_100%_ ≤ 10 Gy	D_100%_ > 10 Gy
		D_max_ ≤ 16Gy	D_max_ ≤ 17 Gy	D_max_ > 17 Gy
OARs constraints	Optic nerves and chiasm	D_max_ ≤ 37.5 Gy	D_max_ ≤ 37.5 Gy	D_max_ > 37.5 Gy
Unscheduled break days	-	0 break days	1–3 break days	> 3 break days

*according to comparison with contours prepared by co-principal investigators.

PTV: planning target volume; D_2%_: dose in 2% of volume; D_98%_: dose in 98% of volume; D_100%_: dose in 100% of volume; D_max_: maximal dose; V_30_: volume irradiated by 30 Gy.

**Figure 3:**
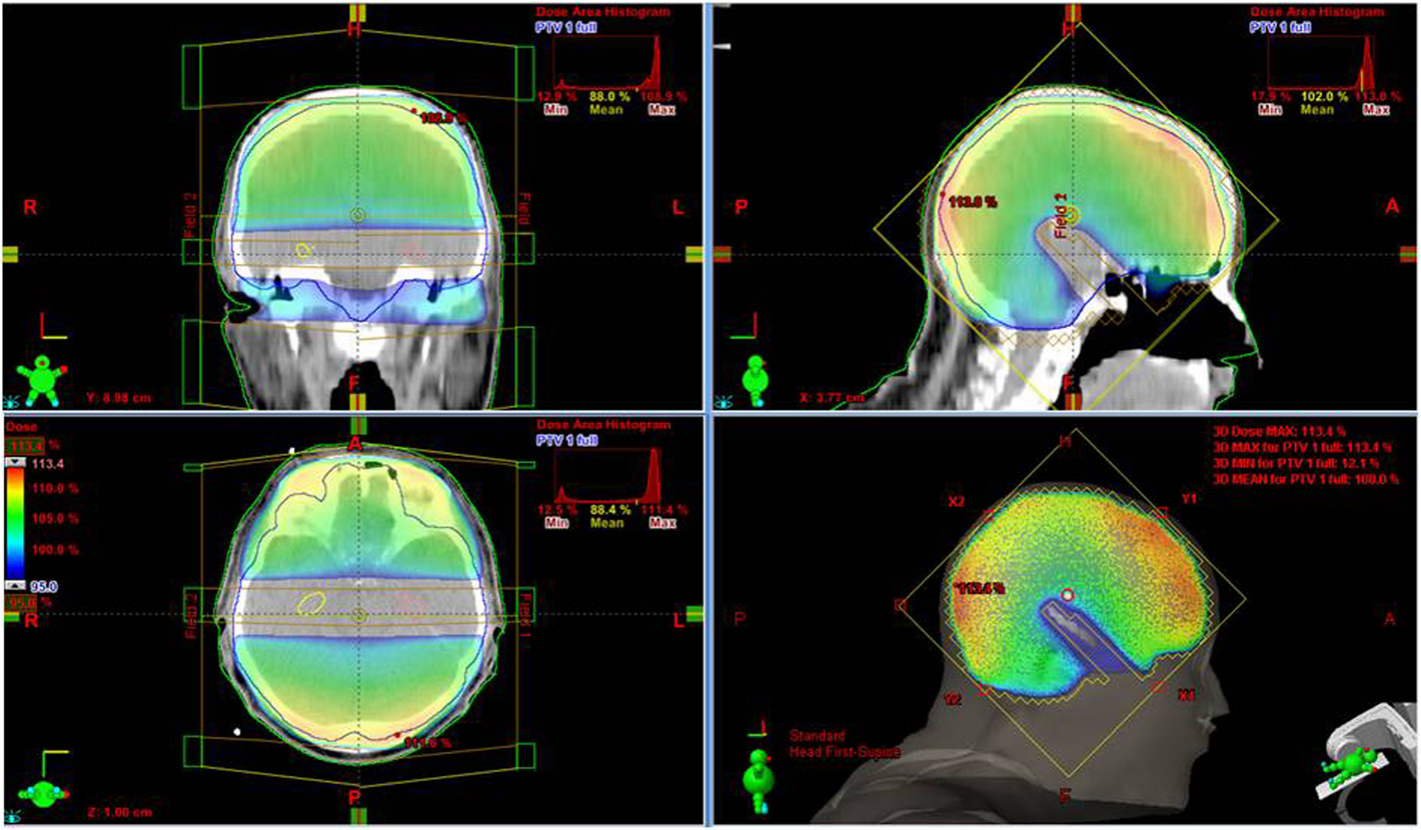
Simple RT technique using 2 laterolateral brain fields with 2 leafs positioned to block the hippocampus.

In addition to the recent sophisticated methods enabling HA during brain irradiation, a simpler technique using two opposing laterolateral fields with central leaf shielding for appropriate HA has also been discussed [39]. The simplicity of this technique could enable radiotherapy departments that are not equipped with the latest technology to still offer HA radiotherapy. This technique is simple reproducible, as demonstrated by one experimental plan from our department (Figure 3).

### Controversies

There are several medical and ethical controversies especially about the indications for HA brain irradiation. Providing of hippocampal sparing techniques is difficult and expensive. Thus, responsible decision must be made with respect to selection of appropriate patients especially in terms of probability of long term survival and QoL. The difference in the cost of basic 3D-CRT and advanced radiotherapeutic methods needed for HA brain irradiation is probably the most important controversy. Unfortunately, in many departments, especially in low-income countries, IMRT techniques are not widely available even for curative treatment (head and neck or prostate cancer). And even in large centers, it is not clear, whether implementation of more expensive RT technique is worthwhile to prevent probable mild neurocognitive decline. Only well designed randomized trials and cost-effective analysis can evaluate whether, or not these approaches should be incorporated into general practice. On the other hand, if IMRT is indicated for another reason, the hippocampus can be considered as other OAR assuming that no other standard OAR will receive more radiation.

Especially for patients with PCI or for children, it is important consider potential AE of some techniques using for hippocampal sparing such as helical tomotherapy for example. In an effort to minimize dose in hippocampus, there is a risk of overtreatment of other parts of the brain and surrounding structures with increased risk of induced secondary malignancies. To assess this potential disadvantage of helical tomotherapy, one study measured integral doses in uninvolved brain regions of HGG patients for both conventional IMRT and helical tomotherapy techniques [57]. The results proved reduction of brain integral dose in average by 23% after IMRT compared with tomotherapy in all tested treatment plans. Despite a theoretical risk of local overtreatment, integral dose delivered by any technique has been surprisingly lower in sparing plans compared with non-sparing ones. From this point of view, usage of traditional IMRT techniques is considered as optimal way how to spare hippocampus. Another approach reducing brain integral dose would be the proton therapy [58].

Considering sparing of some part of brain during WBRT in BM treatment as well as in prophylactic situation, a worry of subsequent increase risk of intracranial disease progression in spared regions is justified. However, many imaging studies described overall low number of metastases in the hippocampus as well as in other parts of the limbic circuit [59-62]. For example, on study evaluated 697 BM in 107 patients, only one of 53 oligometastatic patients (1.9%) had hippocampal metastases (that is 0.97% of all their metastases). In the group of non-oligometastatic patients, in hippocampus was presented only 2.29% of BM [59]. Moreover, other study with 371 patients and 1133 BM localize 8.6% of them into the HA region (hippocampus plus 5 mm margin); however, no metastasis was presented in the hippocampus itself [60]. It can be concluded, that sparing of hippocampus would likely not significantly increase the risk of treatment failure.

On the other hand, others have hypothesized, that neurogenic niches may not only harbor normal NSC but also cancer stem cells responsible for late recurrence. In a retrospective analysis of dose coverage of neurogenic niches in patients with malignant gliomas performed by Evers et al., dose to subventricular zone greater than 43 Gy was associated with a significant improvement in progression free survival compared to those with lower dose (15.0 vs. 7.2 months PFS; P = 0.028) [63]. Interestingly, similar analysis of dose delivered to the hippocampal formation did not yield statistically significant results which confirm the complexity of radiation effects on neurogenic niches [63]. These results highlight the need for well-designed clinical trials as well as continued pre-clinical research to evaluate beneficial or detrimental effects of hippocampal sparing.

The most important treatment related controversy is inconsistency in recommended dose reduction. At this time there is no level I evidence to conclusively support a particular recommendation. Preclinical studies indicate probable no-shoulder dose–response [29]. On the other hand, retrospective clinical studies suggest that biological equivalent dose greater than 7.3 Gy EQD_2_ applied to 40% of hippocampus was associated with worse NCF outcomes [43]. Ongoing phase III trials evaluating NCF function will provide more possible dose-volume constraints associated with possible milder NCF impairment. Because of cost of advanced radiotherapy techniques, it is controversial whether apply intensity modulated plan in some particular patients which will be not able to achieve assumed dose goals especially in situation of standard three dimensional conformal plan would be originally considered. Based on the recently reported phase II trial [11], a dosimetric recommendation for HA WBRT (D_median_ < 7.8 Gy, D100% < 10 Gy and D_max_ < 15.3 Gy) can be recommended for patients with brain metastasis and expected survival greater than 6 months.

### Future directions

Although HA appears to be promising in reducing cognitive affects after RT, ongoing studies and other clinical research are needed to determine the optimal dose and volume constraints. It is not clear based on radiobiology of neural stem cell response to radiation whether it is possible to define specific threshold values in terms of recommended target doses. Even if the nature of radiation injury of hippocampus is same in all patients, the different target doses that can reasonably be achieved in different clinical situations vary with prescribed doses and different clinical target volumes i.e. partial vs. whole brain irradiation or in therapeutic vs. prophylactic indication or in adults vs. children brain tumors. The role of HA in PCI and children has also not been established and is an area of future investigation. Optimal NCF evaluation tools to measure specific effects on hippocampus, as opposed to other etiologies of cognitive dysfunction, and optimal timing of administration is still largely unknown. To be able to compare results from different studies it is necessary to standardize process of NCF testing. However, the ideal tools to measure early changes in cognitive function as compared to late effects of treatment may not be the same. In addition, the testing must be feasible to administer in a busy clinical practice. Ongoing research on pathophysiology of brain irradiation injury may reveal other possible important brain structures whose sparing can contribute to better preservation of NCF, or further analysis of hippocampal subregion (cornu ammonis for example) may demonstrate avoidance region with higher priority for dose sparing. Development of cost-effectiveness analysis will be probably one of the most important steps forward to implementation of this advanced radiotherapy technique especially in low and middle income countries. Comparing cost of standard 3D-CRT (classical 2 latero-lateral fields for WBRT for example) and cost of VMAT or helical tomotherapy systems for example poses important questions whether consequent increase in costs offset theoretical mitigation of neurocognitive decline related to brain irradiation. Especially in situation where are presented many other different sources of cognition impairment in patients suffering from advanced cancer.

## Conclusion

In summary, it is now technically and dosimetrically feasible to implement HA approaches into clinical practice. Furthermore, taking into account very low beam-on time of modern RT systems, it is ethically justifiable to use these techniques also in the palliative indications for patients with BM as well as HGG. As regards boosting of BM, comprehensive techniques with SIB provide tumor doses comparable with sequential approach of classical WBRT + SRS which require even 2 planning procedures which can result in such dosimetric inaccuracies. Moreover, HA WBRT with SIB provides better hippocampal sparing and this treatment approach seems to be the most promising to implement into clinical practice after confirmation of better cognitive outcomes after sparing of hippocampus in ongoing clinical trials.

Recently, first phase II clinical trial with prospectively measured NCF while providing HA WBRT showed significantly better outcomes for patients treated with hippocampal sparing in terms of better cognitive functions as well as quality of life. Conventional techniques of WBRT are now still recommended as standard approach for patients with multiple brain metastases and hippocampal sparing is generally not used outside of the context of clinical trials. Phase III studies are now ongoing and further implementation will depend on the results of these trials. For treatment of PBT, especially in its hemispherical location, it is reasonable to include contralateral hippocampus into the OARs assuming that no other organ at risks or target volumes would be over/under irradiated. Ongoing phase III trials will definitely prove the clinical significance of this developing approach.

## Abbreviations

BT: Brain tumors; PBT: Primary brain tumors; BM: Brain metastasis; PBRT: Partial brain irradiation; HGG: High-grade gliomas; WBRT: Whole brain radiotherapy; SRS: Stereotactic radiosurgery; AE: Adverse effects; NCF: Neurocognitive functions; PCI: Prophylactic cranial irradiation; HA: Hippocampal avoidance; IMRT: Intensity modulated radiotherapy; NSCs: Neural stem cells; OARs: Organs at risks; LINAC: Linear accelerator; VMAT: Volumetric modulated arc therapy; SIB: Simultaneous integrated boost.

## Competing interests

The authors declare that they have no competing interests.

## Authors’ contributions

TK, PP, PS designed the study. TK made the manuscript concept and drafted the article. RJ and NNL provided critical revision and were involved in drafting the manuscript. PB, MS, LH performed the literature search and extracted relevant articles. OS, TP, MV added important content as pictures or tables and included data from their department. All authors participated on revising of the manuscript. All authors read and approved the final manuscript.
